# The Role of N-Glycosylation in Folding, Trafficking, and Functionality of Lysosomal Protein CLN5

**DOI:** 10.1371/journal.pone.0074299

**Published:** 2013-09-10

**Authors:** Akshay Moharir, Sun H. Peck, Theodore Budden, Stella Y. Lee

**Affiliations:** Molecular, Cellular and Developmental Biology Program, Division of Biology, Kansas State University, Manhattan, Kansas, United States of America; Simon Fraser University, Canada

## Abstract

CLN5 is a soluble lysosomal protein with unknown function. Mutations in *CLN5* lead to neuronal ceroid lipofuscinosis, a group of inherited neurodegenerative disorders that mainly affect children. CLN5 has eight potential N-glycosylation sites based on the Asn-X-Thr/Ser consensus sequence. Through site-directed mutagenesis of individual asparagine residues to glutamine on each of the N-glycosylation consensus sites, we showed that all eight putative N-glycosylation sites are utilized *in vivo*. Additionally, localization studies showed that the lack of N-glycosylation on certain sites (N179, N252, N304, or N320) caused CLN5 retention in the endoplasmic reticulum, indicating that glycosylation is important for protein folding. Interestingly, one particular mutant, N401Q, is mislocalized to the Golgi, suggesting that N401 is not important for protein folding but essential for CLN5 trafficking to the lysosome. Finally, we analyzed several patient mutations in which N-glycosylation is affected. The N192S patient mutant is localized to the lysosome, indicating that this mutant has a functional defect in the lysosome. Our results suggest that there are functional differences in various N-glycosylation sites of CLN5 which affect folding, trafficking, and lysosomal function of CLN5.

## Introduction

Neuronal ceroid lipofuscinoses (NCLs), also known collectively as Batten disease, are a group of progressive neurodegenerative disorders that predominantly affect children. NCLs are characterized by mental retardation, impediment of motor capabilities, loss of vision, and premature death [1]. With different ages of onset and progression, several forms of NCL have been characterized as infantile (INCL), late infantile (LINCL), juvenile (JNCL), and adult NCL (ANCL) [2]. NCL is classified as a lysosomal storage disorder based on the buildup of autofluorescent lipopigments, lipofuscin-like ceroids, in the lysosomes of neurons as well as some other cell types [3], [4]. The lipopigments consist of proteins, lipids, and carbohydrates. Depending on the subtype of the disease, the majority of the accumulated proteins are either the subunit C of mitochondrial ATP synthase [5] or saposins A and D [6]. Even though the proteins underlying NCLs are ubiquitously expressed, neuronal cells are by far the most affected cell type by these dysfunctional NCL proteins [7].

*CLN5* is one of the 13 genes that have been identified to be associated with NCLs (NCL resource, University College London). *CLN5* mutations were initially reported to be limited to Finnish and other Northern European populations [8], but a recent study has identified *CLN5* mutations in a variety of ethnic backgrounds [9]. CLN5 disease is mostly associated with the late infantile form of NCLs, although juvenile and adult forms have been identified as well [9], [10]. Human CLN5 consists of 407 amino acids with an N-terminal signal sequence that is cleaved after entering the ER. It does not share any apparent homology with other proteins. CLN5 is a soluble protein [11] despite the presence of a predicted transmembrane segment. It localizes to the lysosomal compartment [11], [12]. The exact function of CLN5 protein is unclear. A recent study reported that CLN5 is essential for the recruitment of retromer, which in turn is responsible for the sorting and recycling of lysosomal receptors [13]. However, this finding is inconsistent with the soluble lysosomal protein properties of CLN5. CLN5 has also been suggested to function as a regulator of dihydroceramide synthase [14], [15].

CLN5 has eight putative N-glycosylation sites based on the consensus sequence of N-X-T/S. Treatment of CLN5 with Endoglycosidase H (Endo H) to remove high mannose type N-linked glycans resulted in a reduction in size from ∼60 kDa to ∼35 kDa, indicating that CLN5 is heavily glycosylated [11]. However, it is not known which of these eight sites are utilized. In another NCL protein, tripeptidyl-peptidase I (TPP I, CLN2 protein), there are five consensus N-glycosylation sites which are all utilized *in vivo* [16]. Of these five, only one residue, N286, seems to have a major effect on its lysosomal localization and maturation processing which subsequently affects enzyme activity. Mutation of this site was identified in some patients (N286S) [17]. Missing glycosylation on this residue causes TPP I to be retained in the ER [16], [18].

Soluble proteins destined to the lysosome generally contain one or more Mannose-6-Phosphate (Man-6-P) residues on their N-linked oligosaccharides. Man-6-P receptors (MPRs) at the trans Golgi network (TGN) recognize these moieties and facilitate sorting and transportation of the Man-6-P tagged proteins to the endosome and subsequently to the lysosome [19]. Interestingly, CLN5 is capable of utilizing MPR-independent pathway(s) to reach the lysosomes in the absence of MPRs [12]. While a proteomic study using mass spectrometry analysis to look for Man-6-P lysosomal proteins has identified Man-6-P sites at N401 and possibly at N320 and/or N330 of human CLN5 [20], there is no definitive data that proves the involvement of these residues in MPR-dependent transport of CLN5.

Three patient mutations in *CLN5* are particularly interesting because they point toward an important role for N-glycosylation in CLN5. One mutant, D279N, introduces a consensus N-glycosylation site, while the other two, N192S and Y392X, lose a potential N-glycosylation site. This prompted us to systematically analyze the importance of CLN5 glycosylation.

In this study, we use site-directed mutagenesis to create mutants for each of the eight predicted N-glycosylation sites and confirm their glycosylation states *in vivo*. We show that all eight consensus sites are used *in vivo*. Loss of N-glycosylation at specific sites leads to mislocalization of CLN5 either to the ER or the Golgi, whereas other mutants show little mislocalization and are able to reach the lysosome.

## Materials and Methods

### Reagents

Cell culture media and reagents were purchased from Gibco and Hyclone. Endoglycosidase H (Endo H_f_), peptide-N-glycosidase F (PNGase F), and other molecular cloning reagents were purchased from New England Biolabs (NEB). The TransIT-LT1 transfection reagent was purchased from Mirus Bio. Cycloheximide was purchased from Fisher Scientific. Tunicamycin was purchased from Enzo Life Sciences. EGFP-Rab5A Q79L was a gift from Qing Zhong (Addgene plasmid 28046; [21]). Sapphire Coomassie Blue kit was obtained from Gold Biotechnology.

### Antibodies

Mouse monoclonal antibodies used in this study were against the Myc epitope (9E10, hybridoma cell line from ATCC (CRL 1729)). Rabbit polyclonal antibodies used in this study were against calnexin (Genscript), Grasp65 (Pierce), and Lamp2 (Pierce). HRP-conjugated secondary antibodies for Western blotting were purchased from Jackson Laboratory. Secondary antibodies conjugated to Alexa Fluor 488, 546, and 633 were purchased from Molecular Probes.

### Site-directed Mutagenesis

The cDNA encoding wild type (wt) CLN5 was purchased from GeneCopoeia and cloned into pcDNA3.1/Myc-His(−)A using EcoRI and BamHI restriction sites. To generate individual N-glycosylation mutants, the codon for Asn in the consensus sequence for N-glycosylation was mutated to a codon for Gln using phusion-based site-directed mutagenesis (NEB). The cDNAs containing the single mutations for the N-glycosylation sites served as templates for creating multiple N-glycosylation mutants. All constructs were confirmed by sequence analysis.

### Cell Culture and Transfections

HeLa cells (ATCC CCL-2) were grown and maintained in Dulbecco’s modified eagle medium (DMEM) supplemented with 10% fetal bovine serum, glutamax, HEPES, and gentamicin at 37^°^C in a humidified incubator with 5% CO_2_. Cells were seeded in a culture dish with or without coverslips 24 h before transfection. Cells were transiently transfected using Mirus TransIT-LT1 transfection reagent according to manufacturer’s protocol. 24 h after transfection, cells were either fixed for immunofluorescence staining or collected for further biochemical analyses. For cycloheximide treatments (50 µg/ml) in immunofluorescence studies, cycloheximide was added to the cells 2 h prior to fixation. For cycloheximide treatment in biochemical studies, cell medium was replaced with Gibco OPTI-MEM reduced serum medium containing 50 µg/ml cycloheximide 20 h after transfection. Cells were collected at 0, 2, and 4 h post treatment, and media were collected at 2 and 4 h post treatment. Medium samples were then concentrated with spin columns (Pierce concentrator, 10 K MWCO) before proceeding to protein gels.

### Immunofluorescence Microscopy

Cells were fixed with 4% formaldehyde for 10 min at room temperature. Blocking, permeabilization, antibody incubations, and washes were done using blocking solution (10% fetal calf serum, 0.1% saponin, and 0.02% sodium azide in PBS). The cells were imaged using a Zeiss LSM-5 PASCAL laser scanning confocal microscope.

### Deglycosylation Experiments

Deglycosylation of samples with Endo H or PNGase F was performed according to manufacturer’s recommendations. Digestion with the enzymes was carried out for 3 h at 37°C. For tunicamycin treatments (1 µg/ml), the chemical was added to cells at the time of transfection.

## Results

### N-glycosylation of CLN5 *in vivo*

**Figure 1:**
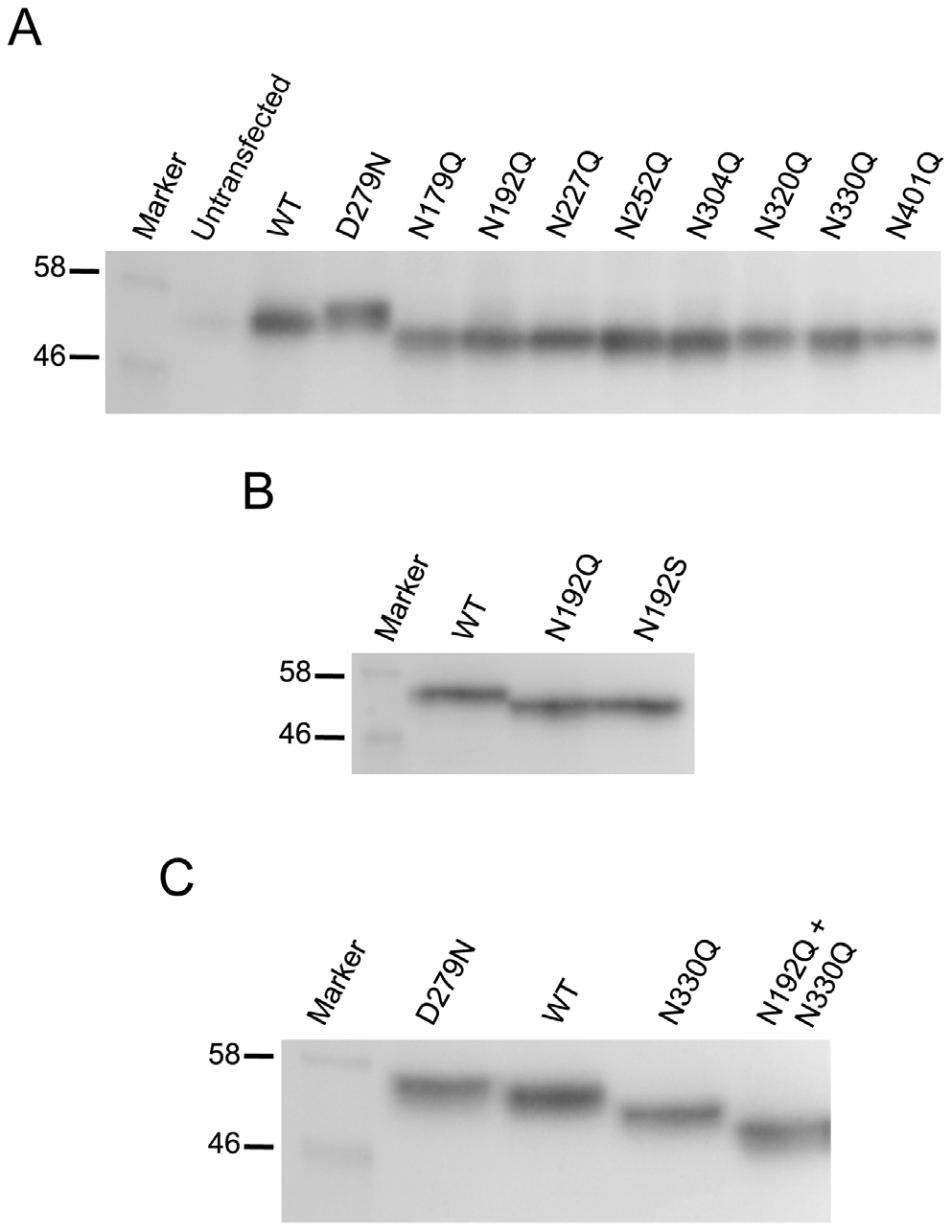
All eight putative N-glycosylation sites of CLN5 are utilized *in vivo*. HeLa cells were transiently expressing the various N-glycosylation mutants of CLN5 as indicated. The whole cell lysates were collected 24 h post transfection and analyzed by Western blotting. (A) wt CLN5, D279N, and eight single N-glycosylation site-deleted mutants as indicated. (B) Comparing N192Q and N192S (patient mutation) migration on gel. (C) Comparing migration on gel of single and double N-glycosylation site mutants. Equal amount of lysates was loaded onto each well. The mouse monoclonal anti-Myc antibody was used to detect CLN5.

Human CLN5 protein consists of 407 amino acids with eight putative N-glycosylation sites located at Asn 179, 192, 227, 252, 304, 320, 330, and 401 (Fig. S1). To determine which of these eight N-glycosylation sites is (are) utilized *in vivo*, we eliminated each of these potential sites in *CLN5* by substituting a Gln codon for the Asn codon. We also recreated a patient mutation D279N [8], which results in an additional N-glycosylation site at residue 279. Wt CLN5 migrated on gel as a species with a molecular weight of ∼55 kDa. Each of the eight N to Q mutants showed an increased mobility in gel corresponding to a ∼2.5 kDa reduction in molecular weight as compared to wt. This shows that each of the eight putative N-glycosylation sites is used *in vivo* (Fig. 1A). The D279N mutant, as has been observed before [12], showed a retarded migration on gel equivalent to a ∼2.5 kDa increase in molecular weight as compared to the wt CLN5. This is consistent with the presence of an additional glycosylation site on the D279N mutant. We also noticed that there were slight mobility differences between the various mutants, which might indicate that not all of the oligosaccharides are modified in an identical fashion. The Western blots were stained with Coomassie blue to show equal sample loading in each lane (Fig. S2).

In addition to mutating the N to a Q for the N192 mutant, we also changed this amino acid to an S to replicate a mutant form that has been isolated from a patient [9]. The N192S showed a similar reduction in size as the N192Q mutant (Fig. 1B), indicating that it is the abrogation of N-glycosylation that changes the mobility of the protein and not some unanticipated behavior by an N to Q mutation in general. Thus, our findings confirmed that all of the eight potential N-glycosylation sites in CLN5 are used *in vivo* and removing any one of these sites results in a reduction of ∼2.5 kDa in size. We also created a double mutant containing both N-glycosylation site mutations of N192Q and N330Q to see if there is indeed an additive effect from the combined mutations. As expected, the double mutant ran ∼2.5 kDa lower than the single mutant and ∼5 kDa lower than wt CLN5 (Fig. 1C).

### Subcellular Localization of CLN5 N-glycosylation Mutants

**Figure 2:**
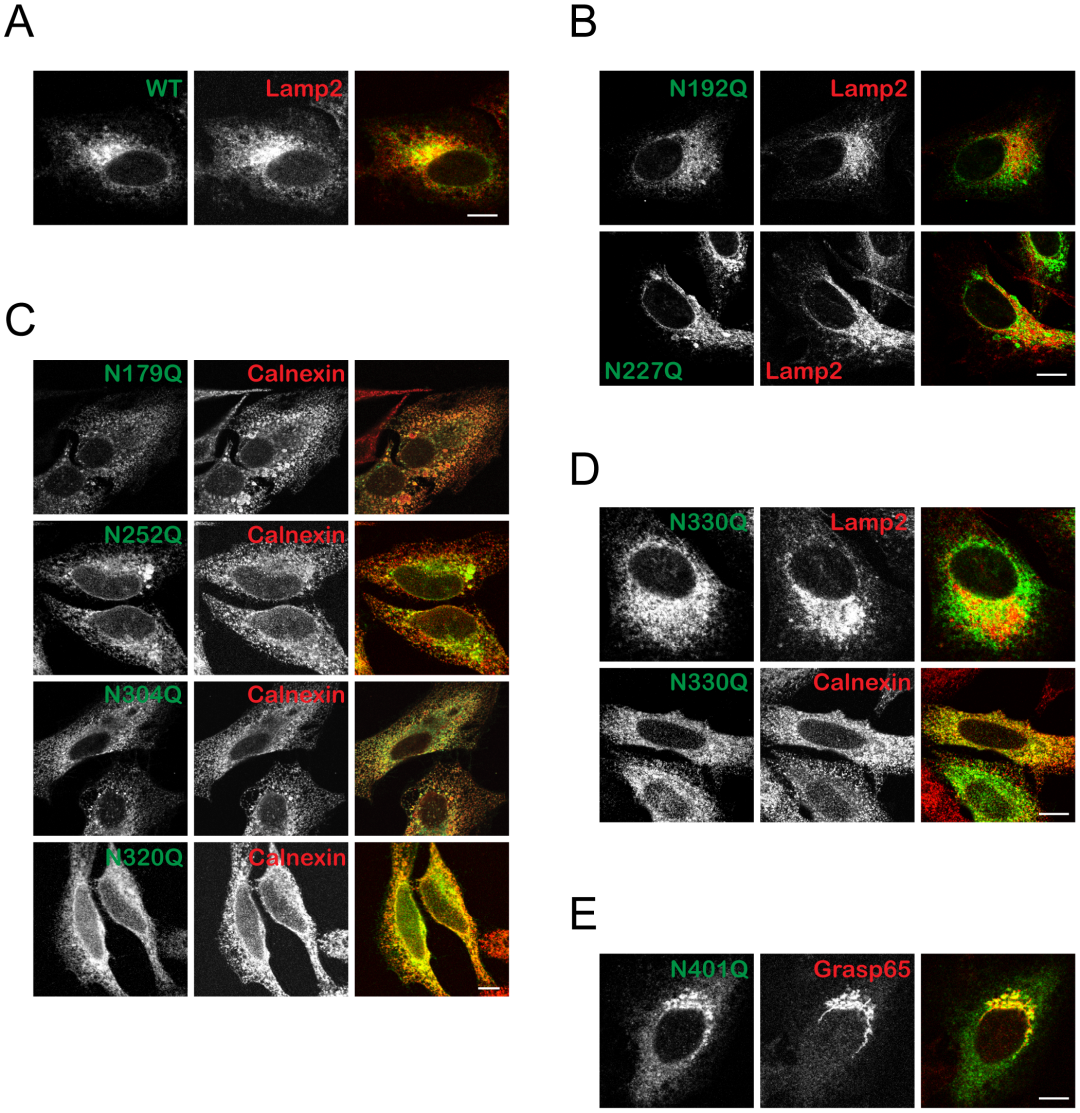
Subcellular localization of CLN5 N-glycosylation mutants. Confocal microscopy analysis of cells transiently expressing N-glycosylation mutants of CLN5. HeLa cells were seeded on glass coverslips and transfected with wt CLN5 or mutants. The cells were treated with cycloheximide for 2 h prior to fixation. Different antibodies were used to label specific organelles (A), (B) and (D) Lamp2 for the lysosomes, (C) and (D) Calnexin for the ER, (E) Grasp65 for the Golgi. Cln5 mutants are as indicated. The mouse monoclonal anti-Myc antibody was used to detect CLN5. Original magnification, 1,000×. Bars, 5 µm.

CLN5 is a lysosomal luminal protein. For proteins localized in the lysosomes, glycosylation can be important for proper folding in the ER, trafficking to the lysosomes, or providing stability and/or functionality in the lysosomes [22], [23]. Thus, if glycosylation on a specific site is crucial for folding, the lack of such glycosylation will result in a misfolded protein that is retained in the ER and targeted for degradation [24]. On the other hand, if a particular glycosylation is essential for targeting the protein to the endosomes and subsequently to the lysosome, the absence of this modification will most likely result in secretion of the protein or accumulation in the Golgi [25]. If the glycosylation mutant can reach the lysosome, it suggests that that specific glycosylation is not critical for folding and trafficking. In such cases, glycosylation might be either redundant or important for the function in the lysosome. Therefore, to assess the function of glycosylation on different sites in CLN5, we examined subcellular localization of each N-glycosylation mutant. HeLa cells were transiently transfected with CLN5. Two hours prior to fixation, cells were treated with cycloheximide to block further protein synthesis in order to ensure minimal amounts of “en route” proteins distorting the analysis. Confocal microscopy showed that the N192Q and N227Q mutants colocalized partially with the lysosomal marker Lamp2, similar to wt CLN5 (Fig. 2, A and B). N179Q, N252Q, N304Q, and N320Q did not colocalize with Lamp2 (data not shown). Instead, these CLN5 mutants colocalized with an ER marker, calnexin (Fig. 2C). N330Q, on the other hand, can be observed in the ER as well as in lysosomes (Fig. 2D). The N401Q mutant colocalized with the Golgi marker, Grasp65 (Fig. 2E). These findings indicate that some of the N-glycosylation sites (N179, 252, 304, 320, and 330) are crucial for the folding of CLN5, as lack of N-glycosylation at any of these sites resulted in CLN5 protein retention in the ER. The N330Q mutation has a milder effect on overall folding, as it partially localized to the lysosome. N401 is essential for trafficking from the Golgi to the lysosome, whereas N192 and N227 seem to have no apparent roles in the proper folding or trafficking of CLN5.

Because wt CLN5 and several mutants partially localized to the lysosome, there might be some ambiguity about the similarity in their localization. Therefore, we decided to manipulate the system by co-expressing different N-glycosylation mutants in combination with a mutant form of Rab5A (EGFP-Rab5A Q79L) [21]. The small GTPase Rab5 gives identity to early endosomes [26] and has been used as an early endosome marker. The mutant Rab5A Q79L has defective GTPase activity, resulting in endosome fusion and the formation of enlarged endosomes [27]. As a consequence, proteins destined to reach the lysosomes will accumulate within these enlarged endosomes (luminal proteins) or on the limiting membrane of the enlarged endosomes (transmembrane proteins).

**Figure 3:**
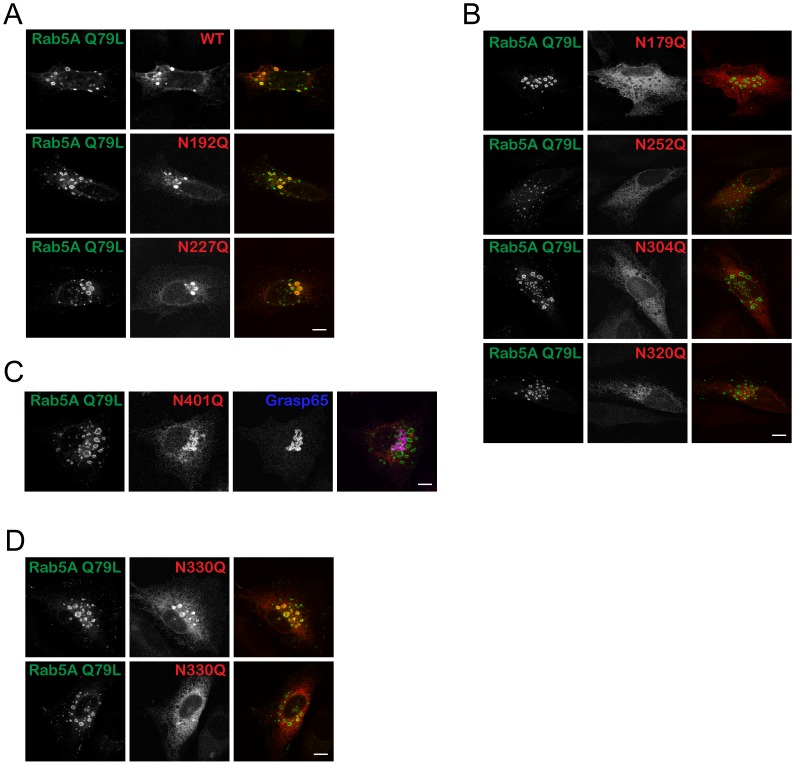
Accumulation of lysosomal-localized CLN5 in Rab5A Q79L-induced enlarged endosomes. HeLa cells were seeded on glass coverslips and double transfected with EGFP-Rab5A Q79L and wt CLN5 or N-glycosylation mutants as indicated. The cells were chased with cycloheximide for 2 h prior to fixation. Localization of (A) CLN5 wt, N192Q, and N227Q, (B) N179Q, N252Q, N304Q, and N320Q, (C) N401, (D) N330Q in Rab5A Q79L expressing cells. Original magnification, 1,000×. Bars, 5 µm.

HeLa cells were co-expressed with EGFP-Rab5A Q79L and wt CLN5 or N-glycosylation mutants followed by 2 h of cycloheximide chase before fixation. The lysosomal mutants (N192Q and N227Q) and wt CLN5 were largely localized inside of the enlarged endosomes (Fig. 3A), indicating that they normally reside in the endosome/lysosome. The ER mutants (N179Q, N252Q, N304Q, and N320Q) did not localize in the proximity of the enlarged endosomes but instead showed a pattern consistent with ER localization (Fig. 3B). N401Q did not localize inside the enlarged endosomes nor did it show a typical ER pattern. When staining for Grasp65, we observed that Golgi morphology was not affected by Rab5A Q79L expression, and the N401Q mutant colocalized with Grasp65 very well (Fig. 3C). N330Q, again, can be seen equally either inside or outside the enlarged endosomes (Fig. 3D). These results are consistent with our earlier colocalization data. Like wt CLN5, N192Q and N227Q mutants can reach the endosome/lysosome, without any ambiguity. However, the majority of the other mutants are either retained in the ER (N179Q, N252Q, N304Q, and N320Q) or accumulated in the Golgi network (N401Q). We noted that a small population of all ER localized mutants escaped from the ER and can be seen in the enlarged endosomes. This is probably due to overexpression of the proteins.

### Deglycosylation Studies of CLN5

**Figure 4:**
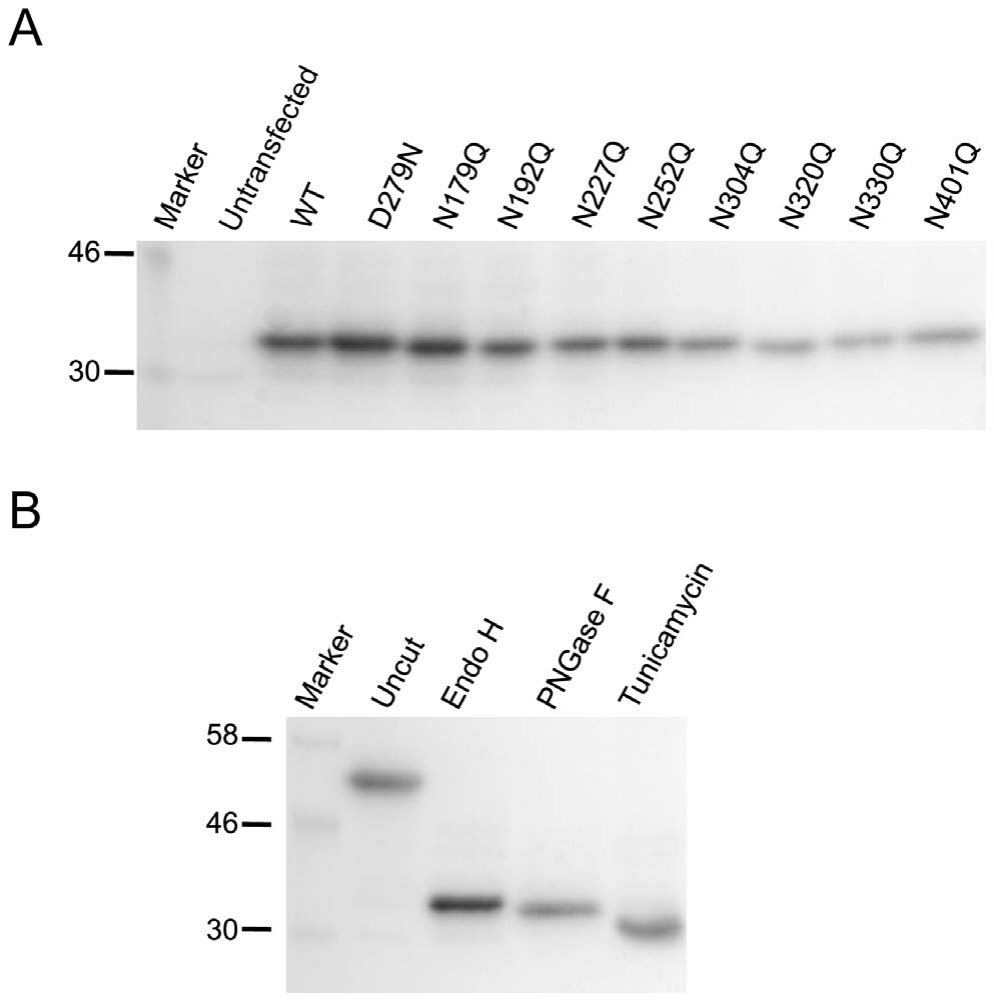
Endo H sensitivity and deglycosylation studies of CLN5. (A) Whole cell lysates transiently expressing N-glycosylation mutants were digested with Endo H enzyme and analyzed by Western blotting. (B) Western blotting of uncut, Endo H, or PNGase F treated whole cell lysates expressing wt CLN5, as well as whole cell lysates from CLN5 transfection in the presence of tunicamycin. Equal amount of lysates was loaded onto each well. The mouse monoclonal anti-Myc antibody was used to detect CLN5.

It has been reported that some glycans on CLN5 are Endoglycosidase H (Endo H) sensitive, and digestion with Endo H results in a reduction of the CLN5 molecular weight [11]. After we digested CLN5 with Endo H, all the single mutants (including D279N) and wt CLN5 migrated on gel to the same size of ∼35 kDa (Fig. 4A). This further confirms that the difference of ∼2.5 kDa between the wt and single glycosylation site mutants was due to N-glycosylation since removal of oligosaccharide chains eliminated the size difference between wt and the mutants. This also implies that all N-glycans on CLN5 are Endo H sensitive, as Endo H digestion on the mutants did not reveal any resistant chains. The difference between the size of undigested wt CLN5 and Endo H digested wt CLN5 was ∼20 kDa (Fig. 1A and 4A), which can be explained by the eight oligosaccharide chains, each of ∼2.5 kDa in size.

To investigate N-glycosylation modification further, we digested CLN5 with peptide N-glycosidase F (PNGase F). Endo H cuts after the first N-acetylglucosamine of the oligosaccharide chain on Asn, thus leaving one sugar moiety on Asn. In contrast, PNGase F cuts immediately after the Asn and does not leave any sugar moiety. When we treated wt CLN5 with PNGase F, we observed faster migration of CLN5 as compared to Endo H treatment, consistent with eight N-acetylglucosamine moieties having been further removed (Fig. 4B). We also examined the effect of tunicamycin treatment. Tunicamycin prevents the first step of N-glycosylation in the ER and as a consequence, the CLN5 synthesized in the presence of tunicamycin will have no glycans attached. Interestingly, we observed an even faster migrating species of CLN5 (∼32 kDa) than those resulting from PNGase F treatment (Fig. 4B). Since tunicamycin treatment causes CLN5 retention in the ER (data not shown), any further modification that takes place in organelles beyond the ER cannot occur.

### Stability of CLN5 Deficient in N-glycosylation

**Figure 5:**
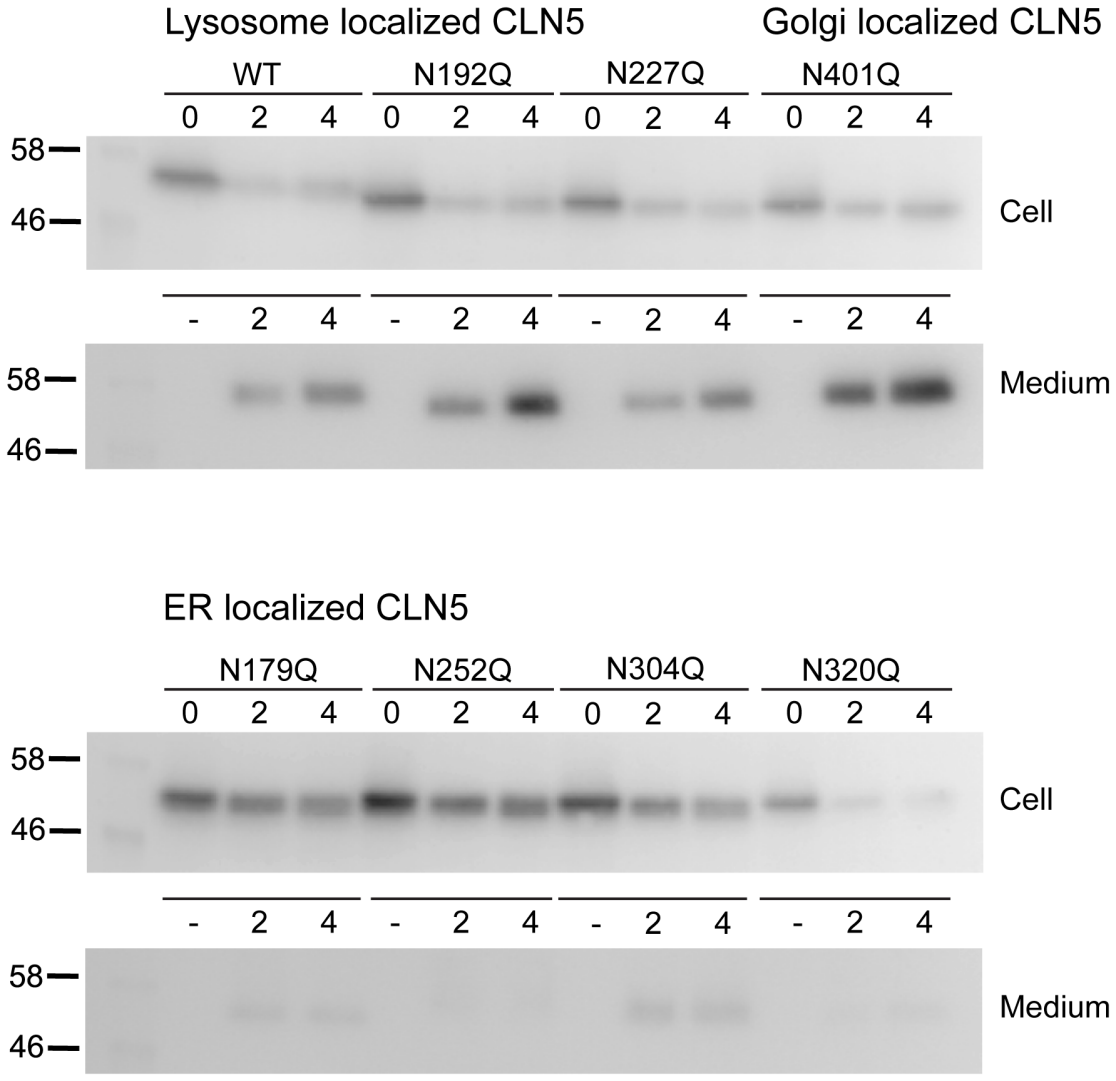
Stability of N-glycosylation deficient CLN5 proteins. HeLa cells were transfected with wt CLN5 or N-glycosylation mutants as indicated for 20 h after which cycloheximide chase was performed for 0, 2, and 4 h. Cell pellets from 0, 2, and 4 h and medium samples from 2 and 4 h are shown. No samples were loaded in lanes labeled (−). The mouse monoclonal anti-Myc antibody was used to detect CLN5.

In our immunofluorescence studies, we noticed that wt CLN5 and mutants that reached the lysosome had lower signal intensity than the ER CLN5 mutants after a 2 h cycloheximide chase. This was surprising, as it could suggest that lysosome-localized CLN5 has a shorter half-life. To test this, we performed transient transfections, replaced the media after 20 h, and directly treated the cells with cycloheximide for 0, 2, or 4 h. Since we, as well as others [11], have detected secreted CLN5 in the media, we also collected the media at 2 and 4 h time points. For the lysosome localized CLN5, the protein levels decrease in the cell pellets with increasing time of cycloheximide chase (Fig. 5, wt, N192Q, and N227Q), consistent with our initial observations of reduced fluorescence. At the same time, we observed a concomitant increase in CLN5 protein levels in the media. Similarly to the lysosome localized CLN5, a large portion of the Golgi localized mutant, N401Q, can be observed in the media after a 4 h cycloheximide chase (Fig. 5, N401Q). This suggests that the reduced fluorescence observed is not due to short half-life or protein degradation, but rather the secretion of CLN5 to the media. This is in agreement with a previous finding that wt CLN5 is stable in cells [12]. In contrast to the lysosome- and Golgi-localized CLN5 proteins, there was much less CLN5 detected in the media for the ER-localized mutants (Fig. 5, N179Q, N252Q, N304Q, and N320Q). All of the ER mutants showed some decrease in protein levels detected in the cell pellet samples over time, but with the lack of significant increase in CLN5 protein levels in the media, it is most likely that the decrease is due to protein degradation in the cell. Quantified representation of this data is shown in Fig. S3.

Based on the localization and stability assays, we can categorize N-glycosylation of CLN5 into three groups (Table 1). In the first group, glycosylation is involved in folding of the protein, without such modification, CLN5 is retained in the ER. In the second group, glycosylation is involved in endosome/lysosome trafficking. Without such signal, CLN5 is accumulated in the Golgi temporarily and then transported towards the plasma membrane for secretion. In the third group, glycosylation does not have a direct role in folding and trafficking but could instead be important for CLN5 lysosomal function or have some redundant role.

**Table 1:** Categorization of CLN5 N-glycosylation sites.

N-glycosylation Residue(s)	Function	Localization(if lacking)
N179, N252, N304, N320, N330*	Folding	ER
N401	Trafficking	Golgi**
N192, N227	Lysosomal function	Lysosome

* some N330Q protein can be observed in the lysosome.

** N401Q mutant is temporarily accumulated in the Golgi and then secreted to the media.

### Subcellular Localization and Stability of CLN5 Patient Mutants

**Figure 6:**
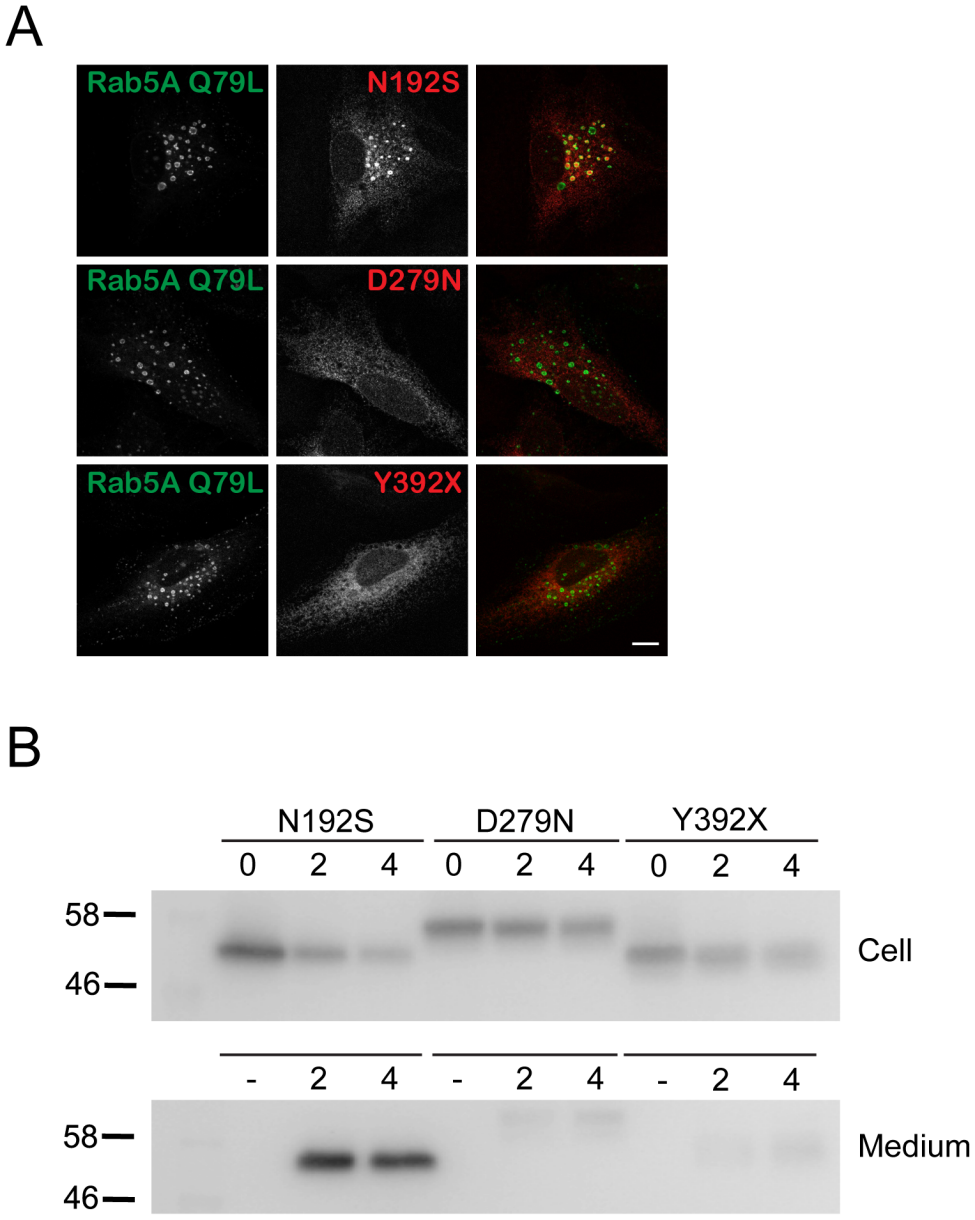
Subcellular localization and stability of CLN5 patient mutations. Confocal microscopy analysis of cells transiently expressing patient mutants of CLN5. (A) HeLa cells were double transfected using EGFP-Rab5A Q79L and D279N, Y392X or N192S. The cells were treated with cycloheximide for 2 h prior to fixation. The mouse monoclonal anti-Myc antibody was used to detect CLN5. Original magnification, 1,000×. Bars, 5 µm. (B) HeLa cells were transfected with patient mutants as indicated for 20 h after which cycloheximide chase was performed for 0, 2, and 4 h. Cell pellets from 0, 2, and 4 h and medium samples from 2 and 4 h are shown. No samples were loaded in lanes labeled (−). The mouse monoclonal anti-Myc antibody was used to detect CLN5.

With the role of CLN5 glycosylation categorized into three functional groups, we now wanted to assign the patient mutants discussed earlier to specific categories. As shown in Fig. 6A, upon co-expression with Rab5A Q79L, the D279N and Y392X mutants did not colocalize with the enlarged endosomes but instead showed localization consistent with presence in the ER. Therefore, we categorize the D279N and Y392X mutants in the group of mutants that cannot fold properly and thus are retained in the ER. As might be expected, based on the N192Q mutant reaching the lysosome, the N192S mutant was able to reach the enlarged endosomes. Thus, the detrimental effect of this mutation is unlikely to be caused by misfolding or improper localization, but most likely due to a functional defect after transportation to the lysosome. This is the first CLN5 patient mutation that has been convincingly shown to localize mostly in the lysosome (see discussion). We also analyzed the possible secretion of these patient mutants using cycloheximide chase and biochemical analyses of cell pellets and media as in Fig. 5 (Fig. 6B). Of the three patient mutants studied, only N192S showed significant protein secretion into the media, which is consistent with the behavior of other lysosome-localized CLN5 mutants. The other patient mutants, which are localized to the ER, remained in the cell pellets. It was additionally notable that the Y392X mutant was less stable than D279N. Quantification data is shown in Fig. S3.

## Discussion

While the function of CLN5 is not known, it is clear that several modifications occur during and after translation. Supported by experimental data [12] and sequence prediction (SignalP 4.1) [28], it is evident that the N-terminus of CLN5 undergoes signal peptide cleavage in the ER during co-translation import (see alignment in Fig. S1). Another major modification is N-glycosylation. In this report, we show that all eight putative N-glycosylation sites of human CLN5 are utilized *in vivo*. Seven of the eight N-glycosylation sites are conserved among mammalian species (orange box in Fig. S1). The last glycosylation site, N401, is not present in *Mus musculus* and *Rattus norvegicus* (green box in Fig. S1). Interestingly, our data demonstrated that N401 glycosylation is essential for lysosomal localization of human CLN5. Without this site, CLN5 is accumulated in the Golgi temporarily and then secreted into the media. This suggests that the Man-6-P modification on N401 is the major determinant of human CLN5 lysosomal transport via MPR-dependent route. Consistent with this, a large-scale proteomic study identified a Man-6-P moiety on N401 of human CLN5 [20]. Besides N401 residue, N320 and N330 of human CLN5 were identified as tentative Man-6-P containing residues [20]. However, our results show that N401Q, with only one glycosylation site removed, was unable to transport to lysosomes, suggesting that either only this site contains a Man-6-P moiety or that other Man-6-P moieties on N320 or N330 are not determinants for MPR-dependent transport for CLN5. N-glycosylation on N320 is essential for proper folding, as N320Q is retained in the ER. Due to retention in the ER of the mutant form, we cannot evaluate the role of N320 in the TGN or endosome trafficking. Even if N320 or N330 residue is tagged with Man-6-Ps in the N401Q mutant, the signal does not suffice for targeting CLN5 to the lysosome because the N401Q mutant is mislocalized.

Intriguingly, using MPRs-deficient MEF cells, mouse and human CLN5 have been shown to reach lysosomes in an MPR-independent manner [12]. As mentioned earlier, mouse CLN5 lacks a residue equivalent to the N401 glycosylation residue found in humans. This may suggest that mouse CLN5 is transported to lysosomes via an MPR-independent pathway. Consistent with this, in mouse CLN5, no specific Man-6-P containing residue was identified [20], [29]. However, mouse CLN5 has still been found in MPR affinity purified cell and/or serum samples in several studies [20], [29], [30]. Based on our findings, it is harder to explain why human CLN5 can also be transported to lysosomes in MPRs-deficient MEF as described in Schmeidt et al. 2010 studies.

From deglycosylation studies, we found that the size of CLN5 synthesized in the presence of tunicamycin is smaller than PNGase F digested CLN5. Three possibilities can potentially explain this. First, there might be a PNGase F resistant glycan on CLN5. However, this is unlikely since PNGase resistant glycans have been found mainly in plants and some insect species [31]. Second, the size difference could be due to polypeptide property changes after PNGase F treatment. This is possible as PNGase F digestion not only removes N-glycans but also modifies the glycan-attached asparagine residue to aspartic acid, whereas tunicamycin treatment does not change CLN5 amino acid residues. The third and most plausible explanation is that there are other modifications on CLN5 besides N-glycosylation. However at this point, we do not know what type(s) of modification causes the molecular weight difference between PNGase F and tunicamycin treated CLN5. Further analysis will be required to address this question.

The localization of patient mutant Y392X has been controversial. The introduction of a premature stop codon results in the elimination of the N401 glycosylation site as well as 15 amino acids. One study showed that the Y392X mutant can reach the Golgi [11], consistent with the lack of N401 glycosylation. However, another study showed that this mutant mainly localizes to the ER [12]. Our immunofluorescence data confirm the latter. This also indicates that the last 15 amino acids have important roles in the overall folding besides containing the N401 dependent lysosomal trafficking signal. Clearly, the ER retention of patient mutant forms Y392X and D279N can explain the lack of functional CLN5 in these patients, as these mutant forms never reach their intended destination. More intriguingly, from a functional perspective, is the N192S patient mutant. Thus far, all other patient mutant forms of CLN5 characterized are mislocalized, including R112P, R112H, E253X, D279N [12] (and our study), L358Afs*4, W379C [32], Y392X [11], [12] (and our study). One study, however, did show D279N and Y392X to be localized to the lysosome, similar to the wt CLN5 [33]. However, the images were over-exposed and all the patient mutants were localized to the lysosome in that study. These findings are inconsistent with the majority of the literature cited above. Here we used several approaches to show that the N192S mutant correctly localizes to the lysosome. Despite correct localization of the CLN5 protein, the N192S mutation (the single mutation in *CLN5* found in this patient) [9] is enough to cause disease manifestation. This suggests that glycosylation on N192 is a crucial element for functionality of the CLN5 protein in the lysosomes. Functional analysis for CLN5 will be needed to further assess the role of glycosylation at this stage.

## Supporting Information

Figure S1 Alignment of mammalian CLN5 protein sequences using CLUSTAL W2 program. The orange boxes with residue number indicate conserved N-glycosylation sites among different species, while the green box indicates the N-glycosylation site corresponding to human N401, which is not conserved in rodents such as *M. musculus* and *R. norvegicus.* The blue dotted line indicates possible cleavage region by signal sequence peptidase. The red asterisks with residue numbers indicate the patient mutants used in this study. Sequences used in this alignment: *H. sapiens* NP_006484, *P. troglodytes* XP_509687, *B. taurus* DAA23821, *C. familiaris* NP_001011556, *M. musculus* AAI41315, and *R. norvegicus* NP_001178618.
(TIF)

Figure S2 Coomassie blue staining of full blots from main Figs. 1, 2, 4, and 6. After immunoblotting, the membranes were stained with Coomassie using the Sapphire Coomassie powder kit (Gold Biotechnology) to show that equal amounts of samples were loaded into each lane.
(TIF)

Figure S3Quantification and normalization of Western blot signals presented in Figs. 5 and 6B. Western blots and Coomassie stained blots (Fig. S2) were imaged using GeneSnap from Syngene and quantified via densitometry using GeneTools analysis software. Quantification used the rolling disk method with a radius of 30 pixels and a Savitsky-Golay filter setting of 3. Samples were normalized against loading densities measured from the Coomassie stained blots as seen in formulas provided below. Cell pellet samples were normalized against protein levels present in the 0 hour time sample of the corresponding set, whereas the medium samples were normalized against the 2 hour time sample within the corresponding set. Values plotted in the graphs represent averages and standard deviations calculated from at least three biologically independent replicates. The y-axis represents arbitrary density units (ADU) as measured by GeneTools. The regions used in Coomassie blot densitometry: cell pellet samples, covering two major bands around 46 KDa; medium samples, covering one major band between 58 and 80 KDa that is present in the OPTI-MEM. Cell pellet samples 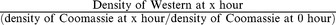 Medium samples 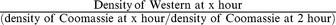.(DOCX)

## References

[1] Zeman W, Dyken P (1969) Neuronal ceroid-lipofuscinosis (Batten’s disease): relationship to amaurotic family idiocy? Pediatrics 44: 570–583. 5346636

[2] Mole SE, Williams RE, Goebel HH (2005) Correlations between genotype, ultrastructural morphology and clinical phenotype in the neuronal ceroid lipofuscinoses. Neurogenetics 6: 107–126. 15965709 10.1007/s10048-005-0218-3

[3] Bennett MJ, Hofmann SL (1999) The neuronal ceroid-lipofuscinoses (Batten disease): a new class of lysosomal storage diseases. J Inherit Metab Dis 22: 535–544. 10407785 10.1023/a:1005564509027

[4] Seehafer SS, Pearce DA (2006) You say lipofuscin, we say ceroid: defining autofluorescent storage material. Neurobiol Aging 27: 576–588. 16455164 10.1016/j.neurobiolaging.2005.12.006

[5] Palmer DN, Fearnley IM, Walker JE, Hall NA, Lake BD, et al (1992) Mitochondrial ATP synthase subunit c storage in the ceroid-lipofuscinoses (Batten disease). Am J Med Genet 42: 561–567. 1535179 10.1002/ajmg.1320420428

[6] Tyynela J, Palmer DN, Baumann M, Haltia M (1993) Storage of saposins A and D in infantile neuronal ceroid-lipofuscinosis. FEBS Lett 330: 8–12. 8370464 10.1016/0014-5793(93)80908-d

[7] Haltia M (2006) The neuronal ceroid-lipofuscinoses: from past to present. Biochim Biophys Acta 1762: 850–856. 16908122 10.1016/j.bbadis.2006.06.010

[8] Savukoski M, Klockars T, Holmberg V, Santavuori P, Lander ES, et al (1998) CLN5, a novel gene encoding a putative transmembrane protein mutated in Finnish variant late infantile neuronal ceroid lipofuscinosis. Nat Genet 19: 286–288. 9662406 10.1038/975

[9] Xin W, Mullen TE, Kiely R, Min J, Feng X, et al (2010) CLN5 mutations are frequent in juvenile and late-onset non-Finnish patients with NCL. Neurology 74: 565–571. 20157158 10.1212/WNL.0b013e3181cff70d

[10] Cannelli N, Nardocci N, Cassandrini D, Morbin M, Aiello C, et al (2007) Revelation of a novel CLN5 mutation in early juvenile neuronal ceroid lipofuscinosis. Neuropediatrics 38: 46–49. 17607606 10.1055/s-2007-981449

[11] Isosomppi J, Vesa J, Jalanko A, Peltonen L (2002) Lysosomal localization of the neuronal ceroid lipofuscinosis CLN5 protein. Hum Mol Genet 11: 885–891. 11971870 10.1093/hmg/11.8.885

[12] Schmiedt ML, Bessa C, Heine C, Ribeiro MG, Jalanko A, et al (2010) The neuronal ceroid lipofuscinosis protein CLN5: new insights into cellular maturation, transport, and consequences of mutations. Hum Mutat 31: 356–365. 20052765 10.1002/humu.21195

[13] Mamo A, Jules F, Dumaresq-Doiron K, Costantino S, Lefrancois S (2012) The role of ceroid lipofuscinosis neuronal protein 5 (CLN5) in endosomal sorting. Mol Cell Biol 32: 1855–1866. 22431521 10.1128/MCB.06726-11 PMC3347407

[14] Schulz A, Mousallem T, Venkataramani M, Persaud-Sawin DA, Zucker A, et al (2006) The CLN9 protein, a regulator of dihydroceramide synthase. J Biol Chem 281: 2784–2794. 16303764 10.1074/jbc.M509483200

[15] Haddad SE, Khoury M, Daoud M, Kantar R, Harati H, et al (2012) CLN5 and CLN8 protein association with ceramide synthase: biochemical and proteomic approaches. Electrophoresis 33: 3798–3809. 23160995 10.1002/elps.201200472

[16] Wujek P, Kida E, Walus M, Wisniewski KE, Golabek AA (2004) N-glycosylation is crucial for folding, trafficking, and stability of human tripeptidyl-peptidase I. J Biol Chem. 279: 12827–12839. 10.1074/jbc.M313173200 14702339

[17] Steinfeld R, Heim P, von Gregory H, Meyer K, Ullrich K, et al (2002) Late infantile neuronal ceroid lipofuscinosis: quantitative description of the clinical course in patients with CLN2 mutations. Am J Med Genet 112: 347–354. 12376936 10.1002/ajmg.10660

[18] Steinfeld R, Steinke HB, Isbrandt D, Kohlschutter A, Gartner J (2004) Mutations in classical late infantile neuronal ceroid lipofuscinosis disrupt transport of tripeptidyl-peptidase I to lysosomes. Hum Mol Genet 13: 2483–2491. 15317752 10.1093/hmg/ddh264

[19] Pohl S, Marschner K, Storch S, Braulke T (2009) Glycosylation- and phosphorylation-dependent intracellular transport of lysosomal hydrolases. Biol Chem 390: 521–527. 19426136 10.1515/BC.2009.076

[20] Sleat DE, Zheng H, Qian M, Lobel P (2006) Identification of sites of mannose 6-phosphorylation on lysosomal proteins. Mol Cell Proteomics 5: 686–701. 16399764 10.1074/mcp.M500343-MCP200

[21] Sun Q, Westphal W, Wong KN, Tan I, Zhong Q (2010) Rubicon controls endosome maturation as a Rab7 effector. Proc Natl Acad Sci U S A 107: 19338–19343. 20974968 10.1073/pnas.1010554107 PMC2984168

[22] Ohtsubo K, Marth JD (2006) Glycosylation in cellular mechanisms of health and disease. Cell 126: 855–867. 16959566 10.1016/j.cell.2006.08.019

[23] Helenius A, Aebi M (2001) Intracellular functions of N-linked glycans. Science 291: 2364–2369. 11269317 10.1126/science.291.5512.2364

[24] Brodsky JL (2012) Cleaning up: ER-associated degradation to the rescue. Cell 151: 1163–1167. 23217703 10.1016/j.cell.2012.11.012 PMC3521611

[25] Bard F, Malhotra V (2006) The formation of TGN-to-plasma-membrane transport carriers. Annu Rev Cell Dev Biol 22: 439–455. 16824007 10.1146/annurev.cellbio.21.012704.133126

[26] Woodman PG (2000) Biogenesis of the sorting endosome: the role of Rab5. Traffic 1: 695–701. 11208157 10.1034/j.1600-0854.2000.010902.x

[27] Stenmark H, Parton RG, Steele-Mortimer O, Lutcke A, Gruenberg J, et al (1994) Inhibition of rab5 GTPase activity stimulates membrane fusion in endocytosis. EMBO J 13: 1287–1296. 8137813 10.1002/j.1460-2075.1994.tb06381.x PMC394944

[28] Petersen TN, Brunak S, von Heijne G, Nielsen H (2011) SignalP 4.0: discriminating signal peptides from transmembrane regions. Nat Methods 8: 785–786. 21959131 10.1038/nmeth.1701

[29] Qian M, Sleat DE, Zheng H, Moore D, Lobel P (2008) Proteomics analysis of serum from mutant mice reveals lysosomal proteins selectively transported by each of the two mannose 6-phosphate receptors. Mol Cell Proteomics 7: 58–70. 17848585 10.1074/mcp.M700217-MCP200

[30] Kollmann K, Mutenda KE, Balleininger M, Eckermann E, von Figura K, et al (2005) Identification of novel lysosomal matrix proteins by proteome analysis. Proteomics 5: 3966–3978. 16145712 10.1002/pmic.200401247

[31] Tretter V, Altmann F, Marz L (1991) Peptide-N4-(N-acetyl-beta-glucosaminyl)asparagine amidase F cannot release glycans with fucose attached alpha 1–3 to the asparagine-linked N-acetylglucosamine residue. Eur J Biochem 199: 647–652. 1868849 10.1111/j.1432-1033.1991.tb16166.x

[32] Lebrun AH, Storch S, Ruschendorf F, Schmiedt ML, Kyttala A, et al (2009) Retention of lysosomal protein CLN5 in the endoplasmic reticulum causes neuronal ceroid lipofuscinosis in Asian sibship. Hum Mutat 30: E651–661. 19309691 10.1002/humu.21010

[33] Vesa J, Chin MH, Oelgeschlager K, Isosomppi J, DellAngelica EC, et al (2002) Neuronal ceroid lipofuscinoses are connected at molecular level: interaction of CLN5 protein with CLN2 and CLN3. Mol Biol Cell 13: 2410–2420. 12134079 10.1091/mbc.E02-01-0031 PMC117323

