# Supplementary figures and images for: The Role of N-Glycosylation in Folding, Trafficking, and Functionality of Lysosomal Protein CLN5
Source: PLoS One. 2013 Sep 10;8(9):e74299. doi: 10.1371/journal.pone.0074299 (PMC3769244; doi:10.1371/journal.pone.0074299)

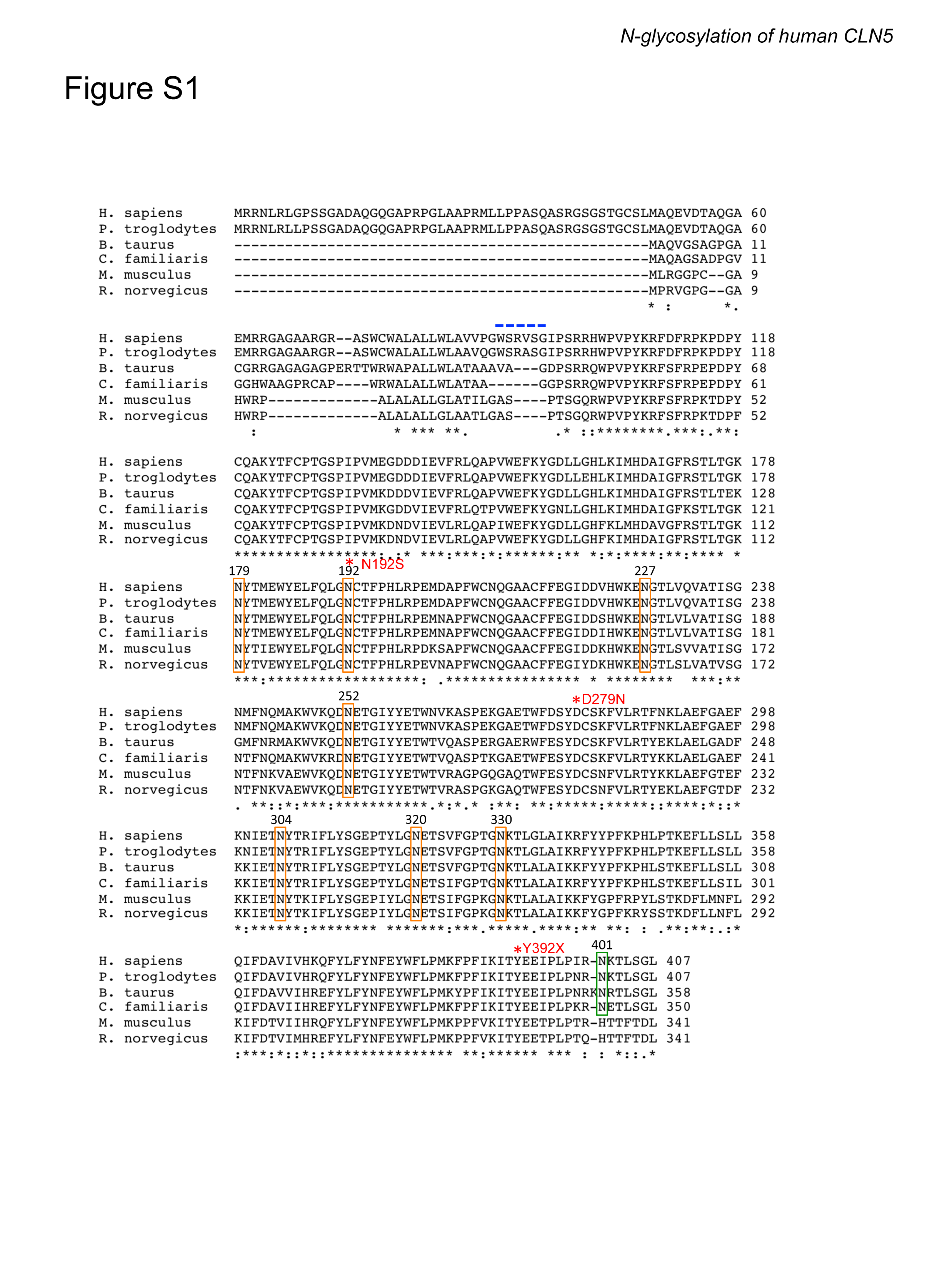

Supplement: Figure S1 — Alignment of mammalian CLN5 protein sequences using CLUSTAL W2 program. The orange boxes with residue number indicate conserved N-glycosylation sites among different species, while the green box indicates the N-glycosylation site corresponding to human N401, which is not conserved in rodents such as M. musculus and R. norvegicus. The blue dotted line indicates possible cleavage region by signal sequence peptidase. The red asterisks with residue numbers indicate the patient mutants used in this study. Sequences used in this alignment: H. sapiens NP_006484, P. troglodytes XP_509687, B. taurus DAA23821, C. familiaris NP_001011556, M. musculus AAI41315, and R. norvegicus NP_001178618. (TIF) [file pone.0074299.s001.tif]

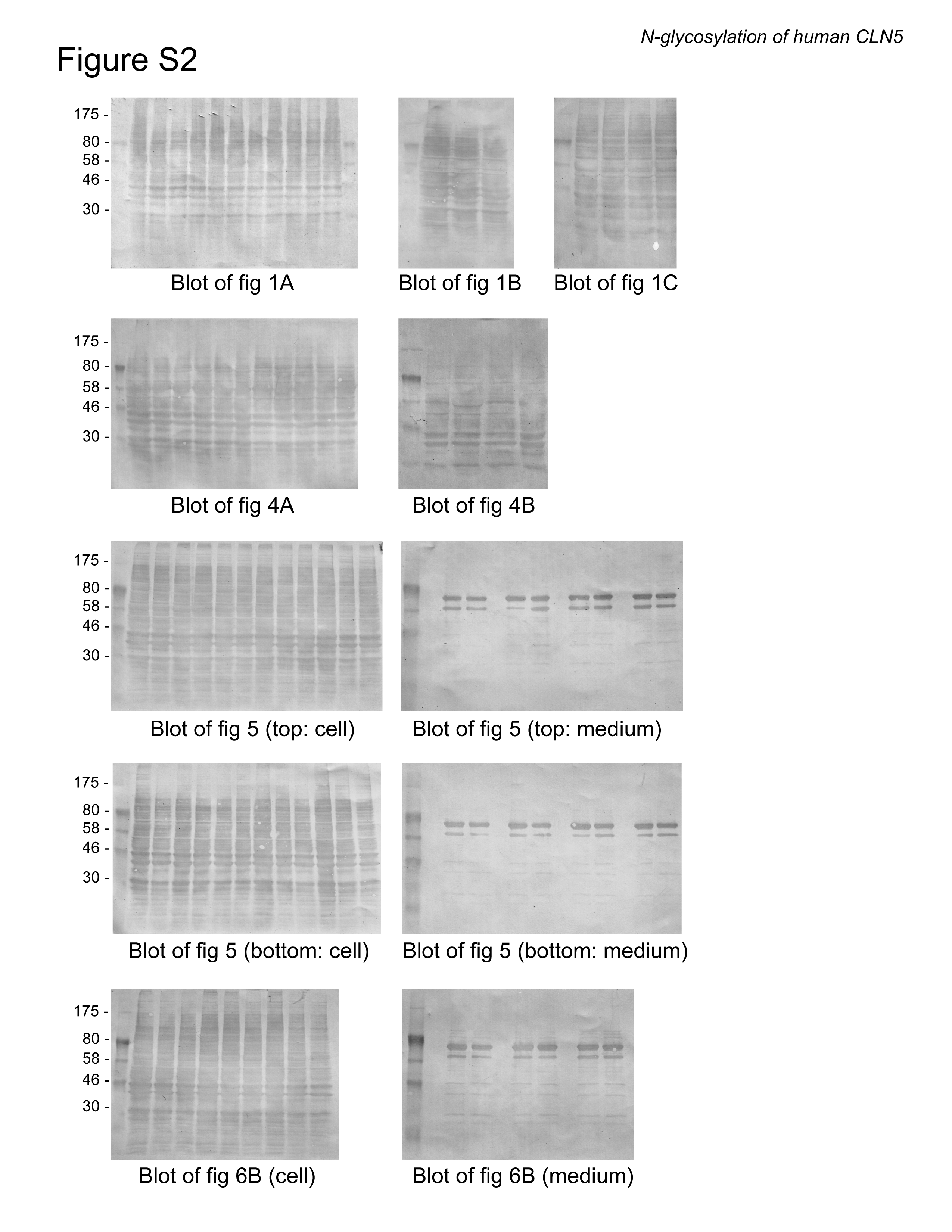

Supplement: Figure S2 — Coomassie blue staining of full blots from main Figs. 1, 2, 4, and 6. After immunoblotting, the membranes were stained with Coomassie using the Sapphire Coomassie powder kit (Gold Biotechnology) to show that equal amounts of samples were loaded into each lane. (TIF) [file pone.0074299.s002.tif]

Figure S3


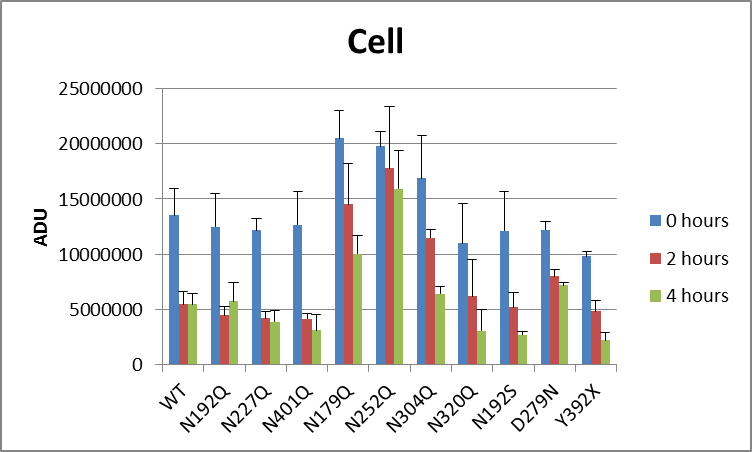


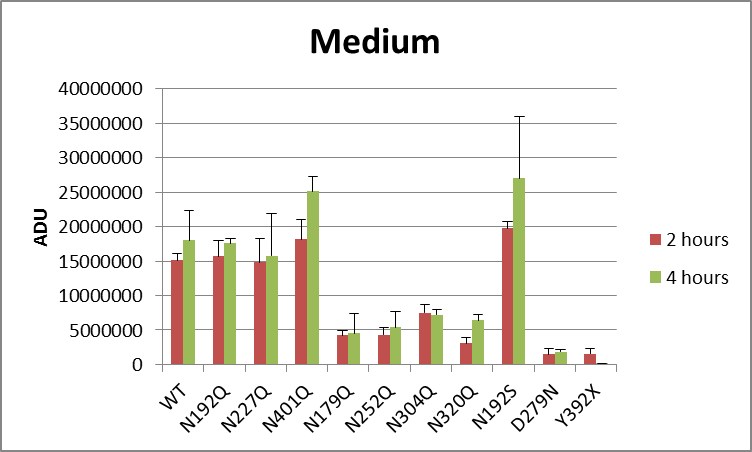

Supplement: Figure S3 — Quantification and normalization of Western blot signals presented in Figs. 5 and 6B. Western blots and Coomassie stained blots (Fig. S2) were imaged using GeneSnap from Syngene and quantified via densitometry using GeneTools analysis software. Quantification used the rolling disk method with a radius of 30 pixels and a Savitsky-Golay filter setting of 3. Samples were normalized against loading densities measured from the Coomassie stained blots as seen in formulas provided below. Cell pellet samples were normalized against protein levels present in the 0 hour time sample of the corresponding set, whereas the medium samples were normalized against the 2 hour time sample within the corresponding set. Values plotted in the graphs represent averages and standard deviations calculated from at least three biologically independent replicates. The y-axis represents arbitrary density units (ADU) as measured by GeneTools. The regions used in Coomassie blot densitometry: cell pellet samples, covering two major bands around 46 KDa; medium samples, covering one major band between 58 and 80 KDa that is present in the OPTI-MEM. Cell pellet samples Medium samples . (DOCX) [file pone.0074299.s003.docx]
